# Hematologic changes under [¹⁷⁷Lu]Lu-DOTA-TATE PRRT in GEP-NETs: a long-term longitudinal cohort study

**DOI:** 10.1186/s13550-026-01543-4

**Published:** 2026-11-10

**Authors:** Christian A. Dascalescu, Felix L. Herr, Ricarda Ebner, Caroline Smith, Victoria Fusch, Matthias P. Fabritius, Mathias J. Zacherl, Florian Eckenweber, Konrad Klimek, Christoph J. Auernhammer, Christine Spitzweg, Maurice M. Heimer, Clemens C. Cyran, Rudolf A. Werner, Gabriel T. Sheikh

**Affiliations:** 1 Department of Radiology, LMU University Hospital, LMU Munich, Marchioninistraße 15, Munich, 81377 Germany; 2 Department of Nuclear Medicine, LMU University Hospital, LMU Munich, Munich, Germany; 3 Department of Medicine IV, LMU University Hospital, LMU Munich, Munich, Germany

**Keywords:** GEP-NET, PRRT, Hematology, Toxicity, Bone marrow

## Abstract

**Background:**

Peptide receptor radionuclide therapy (PRRT) with [¹⁷⁷Lu]Lu-DOTA-TATE is increasingly applied for advanced gastroenteropancreatic neuroendocrine tumors (GEP-NETs). This study aimed to characterize the development of hematologic parameters under PRRT in a large single-center cohort. We retrospectively analyzed 178 patients with GEP-NETs treated with [¹⁷⁷Lu]Lu-DOTA-TATE at a university hospital (2012–2023). Hemoglobin (Hb), white blood cell count (WBC), and platelet count (PLT) were assessed at baseline, after 2 cycles (FU1), after 4 cycles (FU2), during post-therapy follow-up (FU3), and at the last available timepoint (FU4). Patients with deteriorating blood values during or after therapy were categorized according to CTCAE v6.0. The occurrence of myelodysplastic syndrome (MDS) within the period from completion of the initial PRRT to initiation of salvage chemotherapy or PRRT was investigated.

**Results:**

Baseline mean values were Hb, 13.6 ± 1.6 g/dL; WBC, 6.9 ± 2.0 × 10⁹/L; and PLT, 256 ± 92 × 10⁹/L. A significant decline in those hematological parameters was observed at FU1 and FU2 (*p* < 0.01). Between FU2 and FU3, values recovered significantly (*p* < 0.001), with no further relevant change at FU4 for Hb and PLT, while WBC increased significantly compared to FU3. At last follow-up (FU4), all parameters remained significantly below baseline (all *p* < 0.001), but exhibited final values in a normal laboratory range (Hb, 12.4 ± 1.9 g/dL; WBC, 6.3 ± 2.6 × 10⁹/L; and PLT, 216 ± 78 × 10⁹/L). Based on CTCAE v6.0, only 4 patients (2%) developed grade 3 hematological adverse events and no grade 4 adverse event was observed. After completing four cycles of PRRT, 2 patients (1.3%) developed MDS according to the WHO criteria. Patients with chemotherapy prior to PRRT (*n* = 30) had lower baseline WBC and showed smaller on-treatment declines, but were the only subgroup without Hb recovery from the nadir (median change FU2 to FU4, − 0.35 vs. +0.35 g/dL; adjusted *p* = 0.017).

**Conclusion:**

PRRT with [¹⁷⁷Lu]Lu-DOTA-TATE induces a mild short to midterm decline in hematologic parameters, which is followed by a significant increase during long-term follow-up, eventually returning to values within the laboratory reference range. Serious adverse events or MDS are very rare, supporting long-term overall hematologic safety.

## Background

Peptide receptor radionuclide therapy (PRRT) with [^177^Lu]Lu-DOTA-TATE is an effective treatment for somatostatin receptor–positive gastroenteropancreatic neuroendocrine tumors (GEP-NETs), achieving tumor control and survival benefits in patients with advanced disease [1]. The NETTER-1 phase III trial in midgut NETs demonstrated that adding [^177^Lu]Lu-DOTA-TATE to long-acting octreotide significantly prolonged progression-free survival (PFS) and improved quality of life compared to high-dose octreotide alone [1, 2]. Currently PRRT is also being explored in a broader context, including as first-line therapy for higher-grade NETs. Notably, the recent NETTER-2 trial showed that first-line PRRT combined with octreotide in grade 2–3 GEP-NETs showed a highly significant PFS advantage over octreotide alone (median 22.8 vs. 8.5 months) [3, 4]. These advances underscore the growing clinical relevance of PRRT as a cornerstone in NET management and may include a broader range of patients that have been previously not considered eligible for treatment.

A key advantage of PRRT is its generally favorable safety profile. Unlike chemotherapy, PRRT is not typically associated with severe acute toxicity [5–7]. The bone marrow, however, is a critical dose-limiting organ, and thus, there is a clinical need to identify exactly how hematologic values evolve over time. While the randomized prospective NETTER-1 and NETTER-2 trials established the efficacy and overall hematologic safety profile of [¹⁷⁷Lu]Lu-DOTA-TATE based on CTCAE-graded adverse events, they were not designed to comprehensively characterize detailed longitudinal hematological trajectories across extended follow-up [2, 4]. Addressing this knowledge gap could improve patient counselling and ultimately optimize treatment safety and efficacy. Therefore, this study aims to analyze long-term hematological development in GEP-NET patients treated with [^177^Lu]Lu-DOTA-TATE.

## Methods

### General

This retrospective, single-center study was conducted at the LMU University Hospital in Munich, Germany with approval from the local ethics committee (approval number: 25–01311 V), which waived the requirement for informed consent. Clinical, laboratory, and imaging data were collected from patients with histologically confirmed GEP-NETs who received PRRT with [¹⁷⁷Lu]Lu-DOTA-TATE. Eligible patients were 18 years of age or older, had a diagnosis of well-differentiated GEP-NET classified as grade 1 or 2 according to the WHO classification, demonstrated somatostatin receptor (SSTR) expression on SSTR-PET/CT imaging at baseline, and had completed two or four cycles of PRRT with [¹⁷⁷Lu]Lu-DOTA-TATE. The median activity per cycle was 7.4 gigabecquerel (GBq) (range, 3.69–7.65 GBq), adjusted, when necessary, based on individual clinical factors such as renal function. Amino acid infusions were administered in all patients for renal protection during each treatment cycle, as recommended by current guidelines [8, 9]. Treatment was continued to four cycles unless interim restaging or clinical assessment indicated otherwise. Cycles were administered at a median interval of 77 days (interquartile range, 63–91), with interim restaging performed after the second cycle.

### Hematological laboratory assessment

Hematologic parameters, including hemoglobin (Hb), white blood cell count (WBC), and platelet count (PLT), were collected at five time points: baseline (within four weeks prior to the first PRRT cycle), after two treatment cycles (follow-up 1, FU1), after completion of four treatment cycles (follow-up 2, FU2), and during long-term post-therapy follow-up imaging (follow-up 3, FU3), which occurred on average eight months after FU2. In addition, data from a long-term follow-up (follow-up 4, FU4) were collected, representing the last available laboratory assessment prior to initiation of any subsequent therapy, with a median interval of 14.2 months after FU3. Accordingly, to ensure comparability from FU2 onwards, only patients with 4 cycles of PRRT who had not received any other salvage therapy in the interim were included in the analysis. Because FU4 represented the last laboratory assessment available before initiation of any subsequent therapy, the interval between baseline and FU4 varied substantially between patients, with a median of 37.9 months (IQR, 26.9–59.2) and a range from 13.2 to 136.1 months.

Venous blood samples were obtained in EDTA tubes and analyzed in the accredited central laboratory using an automated hematology analyzer as part of routine complete blood count testing. Laboratory abnormalities were interpreted using the institution’s reference ranges. At our center, the lower limits of normal were 11.5 g/dL for women and 13.5 g/dL for men (Hb), 4.00 × 10⁹/L (WBC), and 150 × 10⁹/L (PLT).

Hematologic adverse events were graded according to the Common Terminology Criteria for Adverse Events (CTCAE), version 6.0 [10]. Hemoglobin, leukocyte, and platelet counts were categorized into CTCAE grades at baseline and during follow-up assessments. Treatment-related adverse events were defined as newly occurring or worsening hematologic abnormalities compared to baseline. CTCAE grades that remained unchanged relative to baseline were not considered treatment-related toxicity.

Myelodysplastic syndrome (MDS) was assessed from completion of the initial PRRT course until initiation of any salvage chemotherapy or additional PRRT. MDS was defined according to the World Health Organization (WHO) classification as a clonal hematopoietic stem cell disorder characterized by ineffective hematopoiesis confirmed by bone marrow biopsy, peripheral blood cytopenias, and dysplastic morphology, with an increased risk of progression to acute myeloid leukemia [11, 12].

### Statistics

Descriptive statistics were used to summarize baseline demographic, clinical characteristics, MDS occurrence, as well as hematologic parameters. As hematologic parameters were not normally distributed, longitudinal changes were assessed using paired Wilcoxon signed-rank tests between time points. P-values were adjusted for multiple pairwise comparisons (Benjamini–Hochberg method). For each comparison, analyses were performed on patients with available paired values at the respective two time points and two-sided p-values are reported. Patients with and without prior chemotherapy were compared at each time point using for absolute values, changes from baseline, and recovery from the FU2 nadir the Mann–Whitney U tests, with p-values adjusted per parameter by the Benjamini–Hochberg method. To test whether the trajectory shape differed between groups, linear mixed-effects models (random intercept per patient) were fitted with time point, prior chemotherapy, and their interaction as fixed effects. The interaction was assessed by likelihood ratio test against the main-effects model. Recovery of Hb from the FU2 nadir was further examined in a multivariable linear model adjusted for the FU2 value, age, and sex. All statistical analyses were performed using R (version 4.3.0; R Foundation for Statistical Computing, Vienna, Austria). A p-value of less than 0.05 was considered statistically significant.

## Results

### Patient characteristics

A total of 178 patients with histologically confirmed GEP-NETs who received [¹⁷⁷Lu]Lu-DOTA-TATE were included. The median age was 64 years (range: 33–86), with 61% being male. Baseline characteristics are detailed in Table 1, including tumor grade, primary site and prior therapies. 30 (17%) underwent chemotherapy prior to PRRT, and the specific regimens are detailed in Table 2. Complete laboratory data were available for 178 patients at baseline and FU1, 160 at FU2, 129 at FU3 and 128 at FU4. Within the initial treatment course, 146 patients (82%) received four cycles, 6 (3%) received three cycles, and 26 (15%) received two cycles. Among patients who received three cycles, the reason for treatment discontinuation was not documented in the available records, and treatment continuation outside our institution could not be assessed. Among the 26 patients who received only two cycles, the most common reasons for treatment discontinuation were tumor progression (*n* = 8, 31%), renal dysfunction (*n* = 6, 23%), and hematologic toxicity (*n* = 5, 19%; Table 3).

**Table 1:** Clinical characteristics of study population

	*n* = 178
**Male / female**	108 / 70
**Mean age at baseline.** mean (SD)	63.5 years (10.6)
**Tumor grade**. n (%)	
G1	54 (30%)
G2	119 (67%)
Unknown	5 (3%)
**Primary tumor location**. n (%)	
Small bowel	98 (55%)
Pancreas	37 (21%)
Cancer of Unknown Primary	21 (12%)
Ileocecal junction	11 (6%)
Large bowel / Rectum	9 (5%)
Other	2 (1%)
**Metastases**. n (%)	
Liver	144 (81%)
Lymph nodes	108 (61%)
Mesenterium / Peritoneum	108 (61%)
Bone	105 (59%)
Other	24 (13%)
**Therapy Approach before** PRRT *. n (%)	
Surgical resection of primary tumor	105 (59%)
Long-acting somatostatin analogue	110 (62%)
Local ablative liver therapies	49 (28%)
Chemotherapy	30 (17%) **
Bone-targeted radiotherapy	6 (3%)
* 104 (58.5%) patients received more than one treatment modality before PRRT **5 patients received everolimus in addition to chemotherapy prior to PRRT; no other patient received everolimus before PRRT.	

SD = standard deviation; PRRT = peptide receptor radionuclide therapy; BMI = body mass index; NET = neuroendocrine tumor

**Table 2:** Chemotherapy regimens prior to PRRT

Regimen / drug combination	Patients (*n*)
Capecitabine + Temozolomide	15
Streptozotocin + 5-FU	8
Gemcitabine + 5-FU	4
Regimen not specified	2
Carboplatin + Etoposide	1
FOLFOX	1
Paclitaxel	1
Capecitabine	1
Streptozotocin	1

4 patients received two distinct regimen types, whereas none received three or more distinct chemotherapy regimens prior to PRRT. Regimen not specified indicates that prior chemotherapy was documented, but the exact regimen could not be reliably reconstructed from available records. Of the 30 patients, 23 (76%) had a pancreatic primary, 5 (17%) an intestinal primary and 2 (7%) had cancer of unknown primary (CUP). Chemotherapy data were only partially specified in some cases treated externally, despite extensive review of available medical records; counts are based on the best information retrievable. 5-FU = 5-fluorouracil; FOLFOX = 5-FU/leucovorin/oxaliplatin

**Table 3:** Reasons for discontinuation of PRRT after two cycles

Reason for discontinuation after two cycles	Patients (*n*)	%
Tumor progression	8	30.8
Renal dysfunction	6	23.1
Hematologic toxicity	5	19.2
Mixed response	3	11.5
Reduced somatostatin receptor expression	2	7.7
Poor tolerability	1	3.8
Patient’s decision	1	3.8
**Total**	**26**	**100**

Of 178 patients, 146 (82%) received four cycles within the initial treatment course, 6 (3%) received three cycles, and 26 (15%) received two cycles

### Hb levels

**Fig. 1:**
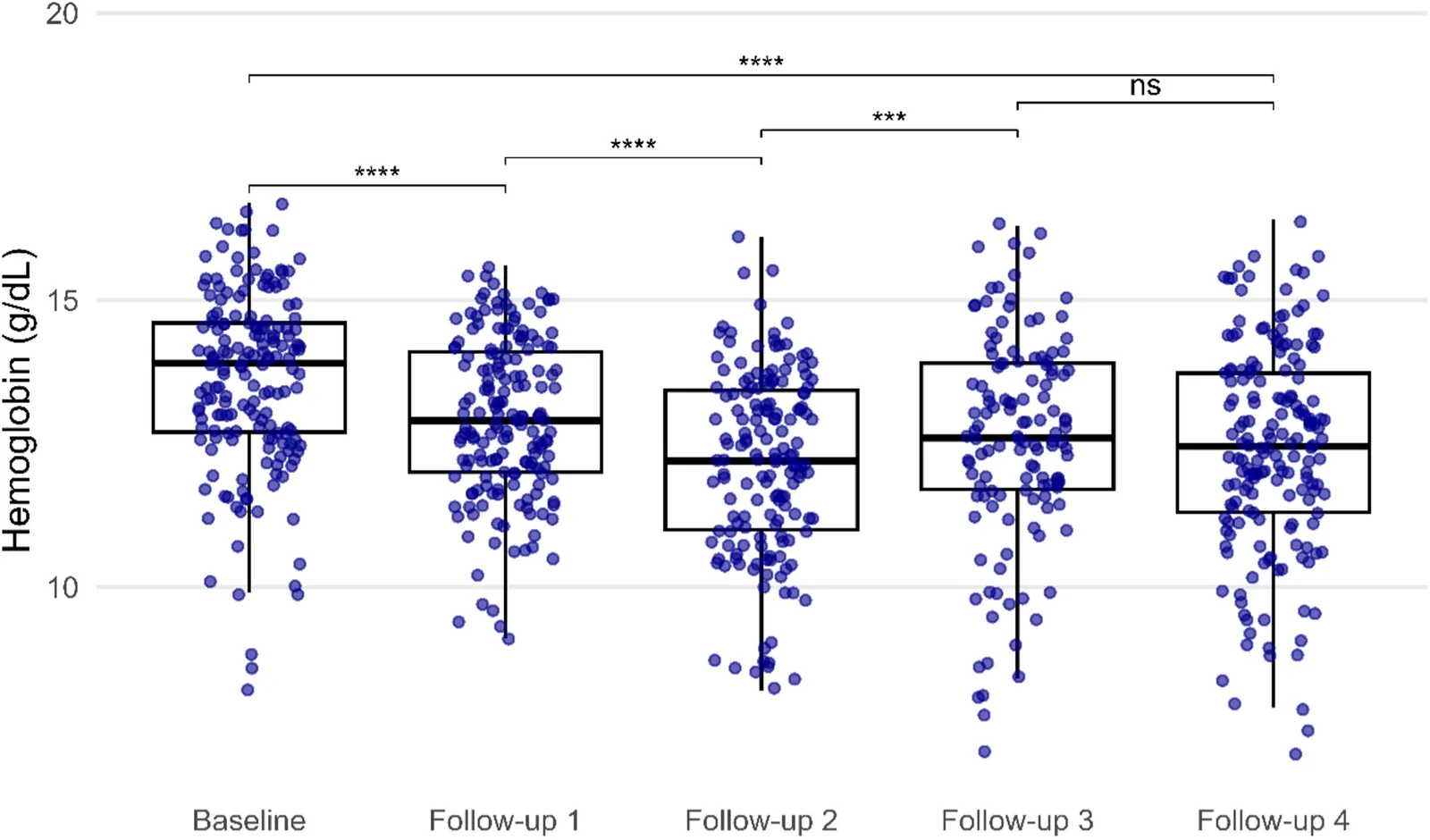
Longitudinal changes in hemoglobin across PRRT treatment and follow-up. Hb declined significantly from baseline to FU1 and FU2, followed by a modest but significant recovery at FU3, with no further relevant change at FU4. Despite remaining below baseline at last follow-up, mean Hb values stayed within the normal laboratory range

Hb declined during PRRT from a mean of 13.6 ± 1.6 g/dL at baseline (median 13.9) to 12.9 ± 1.4 g/dL at FU1 (median 12.9) and reached its lowest values at FU2 (12.2 ± 1.6 g/dL; median 12.2). Across time points, Hb differed significantly overall, and pairwise testing confirmed significant reductions from baseline to FU1 and FU2 (*p* < 0.001, each) and from FU1 to FU2 (*p* < 0.001). At FU3, Hb showed a significant recovery (12.5 ± 1.9 g/dL; median 12.6) compared with FU2 (*p* < 0.001), but remained below baseline (*p* < 0.001). From FU3 to FU4, Hb remained stable without further significant change (12.4 ± 1.9 g/dL; median 12.6; *p* = 0.111), while still significantly lower than baseline (*p* < 0.001) and significantly higher than nadir, i.e., at FU2 (*p* < 0.001; Table 4; Fig. 1).

**Table 4:** Longitudinal changes in hematologic parameters during and after PRRT including median change (Δ) with adjusted p-values from paired Wilcoxon tests

Time point		FU1		FU2		FU3		FU4	
		Δ median	*p*	Δ median	*p*	Δ median	*p*	Δ median	*p*
**Baseline**	Hb	-1	< 0.001	-1.7	< 0.001	-1.3	< 0.001	-1.4	< 0.001
	WBC	-1.8	< 0.001	-2.3	< 0.001	-1.6	< 0.001	-0.7	< 0.001
	PLT	-48	< 0.001	-62	< 0.001	-44	< 0.001	-32	< 0.001
**FU1**	Hb		—	-0.7	< 0.001	-0.3	0.056	-0.4	0.01
	WBC		—	-0.5	< 0.01	0.2	0.210	1.1	< 0.001
	PLT		—	-14	< 0.01	5	0.810	16	0.072
**FU2**	Hb				—	0.4	< 0.001	0.4	< 0.001
	WBC				—	0.7	< 0.001	1.6	< 0.001
	PLT				—	19	< 0.01	30	< 0.001
**FU3**	Hb						—	-0.1	0.111
	WBC						—	0.9	< 0.001
	PLT						—	11	0.160

Significant declines were observed after treatment, with significant increase from nadir at follow-up with levels staying significantly lower than at baseline. Bsl = baseline; FU = follow-up, Hb = hemoglobin; IQR = interquartile range; PLT = platelet count; WBC = white blood cell count. Hb in g/dL; WBC and PLT in ×10⁹/L

### WBC counts

**Fig. 2:**
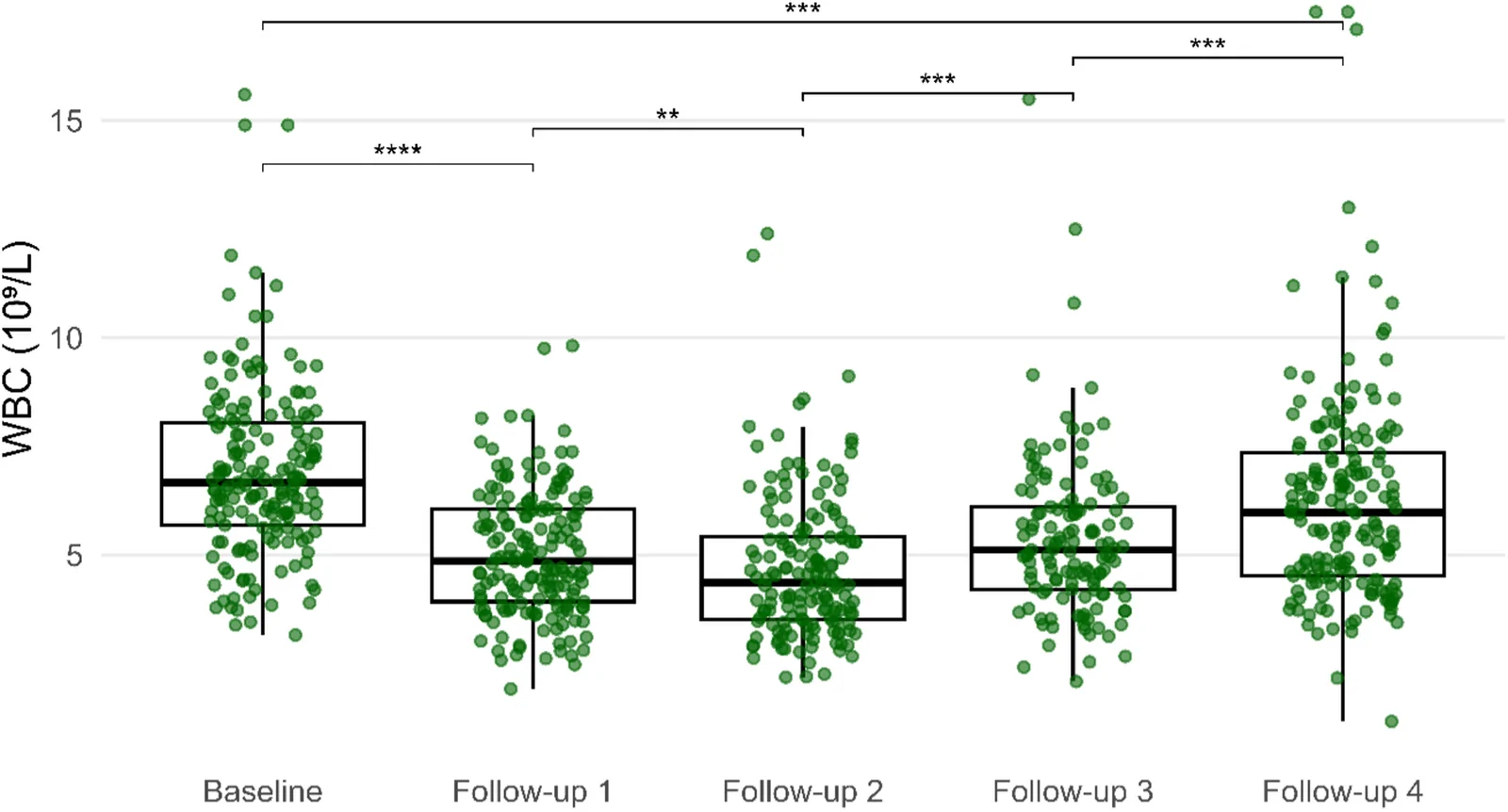
Longitudinal changes in white blood cell count (WBC) across PRRT treatment and follow-up. WBC showed a significant decrease during PRRT (baseline to FU1 and FU2), with significant increase from FU2 to FU3 and at FU4. At last follow-up, WBC remained slightly below baseline but had returned to a normal laboratory range

WBC decreased from a mean of 6.9 ± 2.0 × 10⁹/L at baseline (median 6.7) to 5.0 ± 1.4 × 10⁹/L at FU1 (median 4.9) and further to 4.7 ± 1.7 × 10⁹/L at FU2 (median 4.4), with significant differences between baseline and both on-treatment time points (each *p* < 0.001) and also between FU1 and FU2 (*p* < 0.01). At FU3, WBC increased to 5.4 ± 1.8 × 10⁹/L (median 5.1) compared with FU2 (*p* < 0.001) and was not significantly different from FU1 (*p* = 0.210). At FU4, WBC increased further to 6.3 ± 2.6 × 10⁹/L (median 6.0) and was significantly higher than FU2 and FU3 (both *p* < 0.001), but remained slightly below baseline (*p* < 0.001; Table 4; Fig. 2).

### PLT counts

**Fig. 3:**
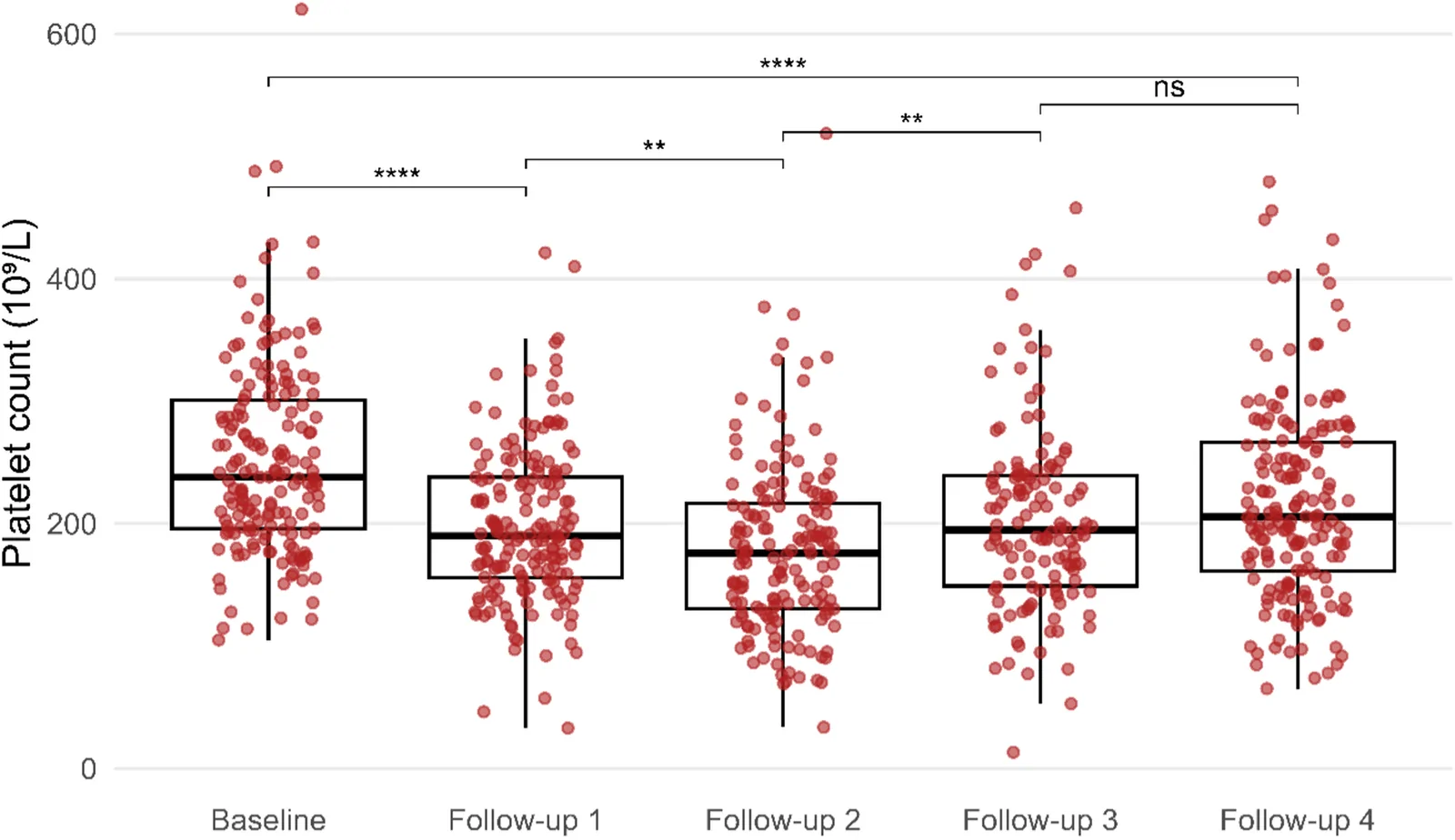
Longitudinal changes in platelet count across PRRT treatment and follow-up. Platelet counts decreased significantly during therapy, reaching the lowest values at FU2, and then increased during follow-up (FU3), remaining stable thereafter (FU4). Although still significantly lower than baseline, platelet levels at last follow-up were within the normal laboratory range

PLT dropped from a mean of 256 ± 92 × 10⁹/L at baseline (median 238) to 198 ± 63 × 10⁹/L at FU1 (median 190) and to 179 ± 70 × 10⁹/L at FU2 (median 176). Pairwise comparisons showed significant decreases from baseline to FU1 and FU2 (both *p* < 0.001) and from FU1 to FU2 (*p* < 0.01). During follow-up, PLT increased to 203 ± 76 × 10⁹/L at FU3 (median 195), which was significantly higher than FU2 (*p* < 0.01), but remained significantly below baseline (*p* < 0.001). At FU4, PLT was 216 ± 78 × 10⁹/L (median 206), showing no significant difference compared with FU3 (*p* = 0.160), but significantly higher values than FU2 (*p* < 0.001), while remaining significantly lower than baseline (*p* < 0.001; Table 4; Fig. 3).

### Impact of prior chemotherapy

Thirty patients (17%) had received chemotherapy before PRRT (Tables 1 and 2). At baseline, these patients had significantly lower WBC than chemotherapy-naïve patients (median 6.02 vs. 6.76 × 10⁹/L; *p* = 0.007, adjusted *p* = 0.034) and numerically lower PLT (208 vs. 242 × 10⁹/L; *p* = 0.031, adjusted *p* = 0.155), whereas baseline Hb was comparable (14.1 vs. 13.8 g/dL; *p* = 0.560). From FU1 onwards, absolute values did not differ significantly between the two groups at any time point after adjustment for multiple comparisons (Table 5, Panel A).

**Table 5:** Hematologic parameters according to chemotherapy prior to PRRT

Parameter	Time point	Prior chemotherapy (*n* = 30)	No prior chemotherapy (*n* = 148)	*p*	*p* (adj.)	
**Panel A — Absolute values, median (IQR) [** ***n*** **]**						
Hb	Baseline	14.1 (12.5–14.7)	13.8 (12.7–14.6)	0.560	0.700	
	FU1	13.6 (13.0–14.3)	12.7 (11.9–13.9)	0.015	0.076	
	FU2	13.2 (11.9–13.6)	12.1 (11.0–13.2)	0.037	0.092	
	FU3	13.2 (11.9–14.1)	12.6 (11.6–13.8)	0.310	0.517	
	FU4	12.9 (10.5–13.6)	12.4 (11.3–13.8)	0.834	0.834	
WBC	Baseline	6.02 (4.20–7.31)	6.76 (5.95–8.12)	0.007	0.034	
	FU1	4.71 (3.69–5.67)	4.89 (3.98–6.13)	0.191	0.477	
	FU2	4.74 (3.35–5.17)	4.34 (3.58–5.54) [133]	0.758	0.860	
	FU3	4.47 (3.65–6.52)	5.14 (4.35–6.05) [113]	0.399	0.665	
	FU4	6.46 (4.17–7.58)	5.98 (4.58–7.63)	0.860	0.860	
PLT	Baseline	208 (155–283)	242 (203–302)	0.031	0.155	
	FU1	202 (137–258)	189 (159–235)	0.801	0.801	
	FU2	193 (114–234)	171 (132–215) [131]	0.571	0.714	
	FU3	238 (182–262)	190 (148–229) [113]	0.090	0.226	
	FU4	239 (190–281)	202 (158–263)	0.247	0.412	
**Panel B — Change from baseline (Bsl)**,** median (IQR) [n]**						
Hb	Bsl → FU1	−0.40 (− 1.20 to 0.60)	−0.85 (− 1.40 to − 0.20)	0.047	0.094	
	Bsl → FU2	−0.75 (− 1.57 to − 0.20)	−1.50 (− 2.28 to − 0.90)	0.005	0.018	
	Bsl → FU3	−0.75 (− 1.00 to − 0.07)	−1.00 (− 1.80 to − 0.30)	0.182	0.243	
	Bsl → FU4	−1.15 (− 2.03 to − 0.48)	−1.20 (− 2.00 to − 0.27)	0.861	0.861	
WBC	Bsl → FU1	−1.14 (− 2.58 to − 0.08)	−1.85 (− 2.70 to − 1.18)	0.013	0.020	
	Bsl → FU2	−1.04 (− 1.80 to − 0.31)	−2.38 (− 3.31 to − 1.33)	< 0.001	< 0.001	
	Bsl → FU3	−0.28 (− 1.77 to 0.24)	−1.74 (− 2.75 to − 0.88)	0.015	0.020	
	Bsl → FU4	0.17 (− 1.56 to 2.10)	−0.84 (− 2.24 to 0.20)	0.060	0.060	
PLT	Bsl → FU1	−25 (− 60 to 20)	−52 (− 81 to − 25)	0.005	0.009	
	Bsl → FU2	−47 (− 68 to − 10)	−77 (− 126 to − 36)	0.002	0.007	
	Bsl → FU3	−28 (− 72 to 30)	−48 (− 103 to − 10)	0.069	0.091	
	Bsl → FU4	−38 (− 61 to 45)	−38 (− 108 to 4)	0.129	0.129	
**Panel C — Recovery from the FU2 nadir**,** median (IQR) [n]**						
Hb	FU2 → FU3	−0.05 (− 0.83 to 0.80)	0.40 (− 0.20 to 1.20)	0.078	0.078	
	FU2 → FU4	−0.35 (− 1.15 to − 0.05)	0.35 (− 0.30 to 1.10)	0.009	0.017	
WBC	FU2 → FU3	0.32 (− 0.49 to 1.47)	0.70 (0.15 to 1.60)	0.289	0.578	
	FU2 → FU4	1.29 (0.50 to 2.86)	1.40 (0.16 to 2.63)	0.763	0.763	
PLT	FU2 → FU3	34 (5 to 62)	23 (2 to 46)	0.466	0.741	
	FU2 → FU4	26 (− 14 to 69)	31 (0 to 64)	0.741	0.741	

Patients who had received chemotherapy before PRRT started with lower WBC and PLT and showed smaller on-treatment declines, whereas recovery from the FU2 nadir was not significantly different between the groups for WBC and PLT except for Hb in previously chemotherapy-treated patients. FU = follow-up; Hb = hemoglobin; IQR = interquartile range; PLT = platelet count; WBC = white blood cell count. Hb in g/dL; WBC and PLT in ×10⁹/L. Panels B and C report patient-level paired changes; differences were calculated for each patient before deriving the median and IQR

Patients with prior chemotherapy showed consistently smaller declines from baseline than chemotherapy-naïve patients. At FU2, the median change from baseline was − 0.75 vs. −1.50 g/dL for Hb (adjusted *p* = 0.018), − 1.04 vs. −2.38 × 10⁹/L for WBC (adjusted *p* < 0.001), and − 47 vs. −77 × 10⁹/L for PLT (adjusted *p* = 0.007; Table 5, Panel B). Linear mixed-effects models confirmed a significant interaction between time point and prior chemotherapy for all three parameters (Hb, χ² = 12.4, df = 4, *p* = 0.015; WBC, χ² = 20.3, df = 4, *p* < 0.001; PLT, χ² = 15.4, df = 4, *p* = 0.004), with all interaction terms indicating a less pronounced on-treatment decrease in previously chemotherapy treated patients. By FU4, the net change from baseline no longer differed between the groups for any parameter (all adjusted *p* ≥ 0.129).

Recovery from the FU2 nadir differed between the groups for Hb only. Between FU2 and FU4, Hb increased by a median of 0.35 g/dL in chemotherapy-naïve patients but decreased by 0.35 g/dL in patients with prior chemotherapy (adjusted *p* = 0.017; Table 5, Panel C). This difference persisted after adjustment for the FU2 value, age, and sex (β = −1.00 g/dL; 95% CI, − 1.77 to − 0.24; *p* = 0.010). In contrast, recovery of WBC and PLT was comparable between the groups (all adjusted *p* ≥ 0.741). Neither of the two patients who developed MDS had received chemotherapy prior to PRRT.

### Adverse events and MDS

In 39 patients (22%), at least one hematological parameter deteriorated to ≥ CTCAE v6.0 grade 2 during follow-up, of whom only 4 patients (2%) developed grade 3 toxicity. No grade 4 toxicity occurred in the cohort. After completing four cycles of PRRT, 2 patients (1.3%) developed biopsy-confirmed MDS before initiation of any subsequent salvage therapy. The time from PRRT completion to diagnosis by bone marrow biopsy was 28 and 53 months, respectively. Regarding treatments received, neither of these patients underwent chemotherapy or received everolimus before PRRT or during the period up to MDS diagnosis. In one case, PRRT was preceded by seven years of somatostatin analogue (SSA) therapy; in the other case, PRRT was initiated shortly after diagnosis as the first systemic treatment. Following completion of PRRT, both patients continued SSA maintenance with lanreotide 120 mg every 4 weeks until MDS diagnosis.

## Discussion

In this study, we characterized the development of hematologic parameters and recovery timeline in GEP-NET patients undergoing PRRT. All three blood parameters, Hb, WBC and PLT, showed a significant decline during treatment. After two and four PRRT cycles, counts had dropped from baseline values, while a significant hematologic recovery was observed after completion of PRRT. However, despite an observed rebound, values did not fully return to pre-treatment baselines on average, as all three metrics stayed slightly below their baseline value. Nonetheless, at last available follow-up, recorded average values were still within normal laboratory ranges (Hb, 12.4 ± 1.9 g/dL; WBC, 6.3 ± 2.6 × 10⁹/L; and PLT, 216 ± 78 × 10⁹/L). Importantly, severe hematologic toxicity and therapy-related myelodysplastic syndrome were very rare in this cohort. As such, despite a mild persistent decrement in hematopoietic parameters, patients generally seem to recover well from the hematological impact of PRRT.

Our findings illuminate a clear recovery trajectory after primary decrease of hematologic values. The pattern, acute declines during treatment followed by partial recovery within the first year, is consistent with prior reports. For instance, Sabet et al. observed that blood counts typically normalized about 12 months after PRRT on average (range 3–22 months) [13]. Also NETTER-1 demonstrated the efficacy of PRRT [¹⁷⁷Lu]Lu-DOTA-TATE with a clinically significant decrease of hematological values occurring in less than 10% of treated patients [2]. Similarly, Kwekkeboom et al. described relevant hematologic decrease occurring only in 3.6% after each administration. The declines (when present) were subacute (occurring approximately 4–8 weeks after administration) and temporary, followed by later recovery rather than progressive deterioration [14]. Similarly, Bergsma et al. reported that hematologic values show a subacute nadir typically approximately 4–6 weeks after each administration, followed by a recovery phase [15]. This reinforces earlier conclusions that PRRT-related myelosuppression rarely necessitates dose adjustment or cessation [13].

In NETTER-1, grade 3 or 4 lymphopenia, thrombocytopenia, and anemia occurred in 9%, 2%, and 0% of patients treated with [¹⁷⁷Lu]Lu-DOTA-TATE, respectively [2]. In NETTER-2, in which patients with grade 2–3 GEP-NETs received first-line PRRT, grade 3 or 4 leukopenia, thrombocytopenia and anemia were each reported in fewer than 3 of 147 treated patients (≤ 2%) [4]. In the large Erasmus MC series, subacute grade 3 or 4 hematologic toxicity occurred in 61 of 582 patients (10%), comprising leukocyte toxicity in 5%, thrombocytopenia in 5%, and hemoglobin toxicity in 4% [5]. Kwekkeboom et al. similarly reported subacute grade 3 or 4 hematologic toxicity in 9.5% of 504 patients [14]. The grade 3 rate of 2% and the absence of grade 4 events in our cohort therefore lie at the lower end of the published range, acknowledging that our assessment was based on scheduled restaging time points rather than per-cycle nadirs. Transient nadirs expected approximately 4 to 6 weeks after individual administrations [15] may therefore not have been captured, leading to a possible underestimation compared to published data. The extended observation period of the present study did, however, allow characterization of the late hematologic trajectory, which was not explicitly reported in the abovementioned studies.

While the WBC and PLT showed an overall increase after FU2 at late follow-up, the recovery of Hb increase was less pronounced. Persistent post-PRRT anemia could also plausibly be linked to inflammation-driven iron restriction (functional iron deficiency), that can suppress Hb despite recovery of other lineages [16]. Moreover, older age (median age at baseline = 64 years) may be associated with a less pronounced Hb recovery, reflecting reduced hematopoietic reserve and a greater comorbidity burden [15, 17]. Baseline anemia, prior cytotoxic/alkylating therapy, or nutritional deficits may further impair Hb normalization [18, 19]. Our subgroup analysis supports this interpretation. Although patients with prior chemotherapy showed smaller absolute declines during PRRT, largely because their baseline WBC and PLT were already lower, they were the only subgroup in which Hb failed to recover from the FU2 nadir, and this difference persisted after adjustment for the nadir value, age, and sex. Alkylating and platinum-containing regimens, which accounted for the majority of pretreatments in our cohort (Table 2), are known to cause long-lasting damage to the erythroid compartment, and the additional radiation dose delivered to the bone marrow during PRRT may further limit the residual capacity for erythroid regeneration. Because only 16 previously chemotherapy-treated patients contributed data at FU3 and FU4, this observation should be regarded as hypothesis-generating.

The clinical implications of these results are significant for patient management and monitoring. Knowing that blood counts reach their lowest point at the end of PRRT and then improve, clinicians can better time follow-up evaluations and supportive care. Therefore, close hematological monitoring during therapy and in the months following PRRT is recommended. The described recovery timeline is also important when considering subsequent therapies. For instance, if a patient may benefit from salvage PRRT or chemotherapy upon disease progression, allowing a treatment-absent time window for marrow recovery (when feasible) could mitigate compounded suppression of hematological function. Our data suggest that the bone marrow can recuperate sufficiently to permit further treatments in the majority of patients and that long-term bone marrow toxicity is rare.

This is reflected in the low incidence of MDS observed in our cohort (2 cases among patients who completed four cycles PRRT, 1.3%) and comparable to published literature. For example, Bodei et al. noted approximately 2.3% of patients developing MDS after PRRT in a cohort of 807 subjects, consistent with other studies that generally report < 3% of patients experiencing this complication [20, 21]. MDS is thought to be multifactorial, influenced by prior or subsequent chemo-radiotoxic therapies, as well as intrinsic predispositions such as clonal hematopoiesis [22]. Of note, the 2 cases in our cohort had not received respective chemotherapies that are known for being associated with increased risk of MDS, while other factors such as clonal hematopoiesis of indeterminate potential may have also contributed to the development of MDS [22, 23]. In this regard, prospective designs could address pre-existing hematologic mutations or precursor conditions (e.g. clonal hematopoiesis of indeterminate potential), which are now recognized as risk factors for myeloid neoplasms, including MDS or further hematooncologic diseases. Bodei et al. have also identified associations between MDS development and factors like high-grade thrombocytopenia during therapy and a prolonged PRRT course, while the low number of MDS events in our series was too low to permit any meaningful risk factor analysis [20]. Therefore further research is needed to investigate possible causes for MDS occurrence, preferably by larger cohort in a multicenter setting [22, 23].

Several limitations of this study must be acknowledged. First, this was a retrospective single-center study, which limits generalizability, in particular as we cannot rule out that laboratory analyses procedures changed over time. Although conducted at a high-volume center, institutional practices in monitoring may differ from other institutions. Second, this study was designed to characterize longitudinal laboratory trajectories of Hb, WBC, and PLT and clinical adverse events were not analyzed (e.g., bleeding, infections, transfusions, dose delays). Nevertheless, describing the expected course of hematologic parameters across treatment and follow-up provides a practical reference for patient counseling and for structuring laboratory monitoring in clinical routine. Future prospective studies with extended follow-up and larger number of patients are warranted to better capture late hematologic trends. Finally, the subgroup of patients who had received chemotherapy prior to PRRT was small (*n* = 30) and became smaller during long-term follow-up (*n* = 16 at FU3 and FU4), which limits the precision of the subgroup comparisons and precludes analysis of individual regimens. Patients selected for chemotherapy before PRRT also differed systematically from chemotherapy-naïve patients, with a higher Ki-67 index (median 10% vs. 4%; *p* < 0.001) and a predominance of pancreatic primary tumors, so residual confounding by indication cannot be excluded.

## Conclusion

Overall, we herein demonstrate a predictable temporal pattern of Hb, WBC, and PLT changes, characterized by an initial decline during therapy, followed by a nadir and recovery during follow-up. Of note, all three parameters exhibited values in normal laboratory ranges upon latest follow-up and severe hematologic adverse events or MDS during or after PRRT proved to be very rare. These findings from one of [¹⁷⁷Lu]Lu-DOTA-TATE largest cohorts evaluated so far complement existing PRRT literature and support structured laboratory monitoring across treatment and into longer-term follow-up.

## Data Availability

The datasets generated and/or analyzed during the current study are not publicly available due to institutional data protection regulations and the retrospective use of patient-related clinical data, but may be available from the corresponding author upon reasonable request and subject to approval by the responsible institutional authorities.

## Abbreviations

5-FU: 5-fluorouracil
AML: Acute myeloid leukemia
BH: Benjamini–Hochberg
BMI: Body mass index
CBC: Complete blood count
CUP: Cancer of unknown primary
CTCAE: Common Terminology Criteria for Adverse Events
EDTA: Ethylenediaminetetraacetic acid
ENETS: European Neuroendocrine Tumor Society
FU: Follow-up
FU1: Follow-up after two PRRT cycles
FU2: Follow-up after four PRRT cycles
FU3: Post-therapy follow-up
FU4: Last available follow-up prior to subsequent therapy
GBq: Gigabecquerel
GEP-NET: Gastroenteropancreatic neuroendocrine tumor
Hb: Hemoglobin
LMU: Ludwig-Maximilians-Universität
MDS: Myelodysplastic syndrome
NET: Neuroendocrine tumor
PFS: Progression-free survival
PLT: Platelet count
PRRT: Peptide receptor radionuclide therapy
SD: Standard deviation
SSA: Somatostatin analogue
SSTR: Somatostatin receptor
WBC: White blood cell count
WHO: World Health Organization

## References

[1] de Vries-Huizing DMV, Versleijen MWJ, Sinaasappel M, Walraven I, Geluk-Jonker MM, Tesselaar MET, et al. Haematotoxicity during peptide receptor radionuclide therapy: Baseline parameters differences and effect on patient’s therapy course. PLoS ONE. 2021;16(11):e0260073. 10.1371/journal.pone.0260073 PMC8601524 34793530

[2] Strosberg J, El-Haddad G, Wolin E, Hendifar A, Yao J, Chasen B, et al. Phase 3 Trial of (177)Lu-Dotatate for Midgut Neuroendocrine Tumors. N Engl J Med. 2017;376(2):125–35. 10.1056/NEJMoa1607427 PMC5895095 28076709

[3] Highlights in neuroendocrine tumors from the 2024 North American Neuroendocrine Tumor Society (NANETS) Multidisciplinary NET Medical Symposium. Clin Adv Hematol Oncol. 2024;22 Suppl 8(10):1–16. 39688624

[4] Singh S, Halperin D, Myrehaug S, Herrmann K, Pavel M, Kunz PL, et al. [(177)Lu]Lu-DOTA-TATE plus long-acting octreotide versus high–dose long-acting octreotide for the treatment of newly diagnosed, advanced grade 2–3, well-differentiated, gastroenteropancreatic neuroendocrine tumours (NETTER-2): an open-label, randomised, phase 3 study. Lancet. 2024;403(10446):2807–17. 10.1016/S0140-6736(24)00701-3 38851203

[5] Brabander T, van der Zwan WA, Teunissen JJM, Kam BLR, Feelders RA, de Herder WW, et al. Long-Term Efficacy, Survival, and Safety of [(177)Lu-DOTA(0),Tyr(3)]octreotate in Patients with Gastroenteropancreatic and Bronchial Neuroendocrine Tumors. Clin Cancer Res. 2017;23(16):4617–24. 10.1158/1078-0432.CCR-16-2743 28428192

[6] Riesco-Martinez MC, Capdevila J, Alonso V, Jimenez-Fonseca P, Teule A, Grande E, et al. Nivolumab plus platinum-doublet chemotherapy in treatment-naive patients with advanced grade 3 Neuroendocrine Neoplasms of gastroenteropancreatic or unknown origin: The multicenter phase 2 NICE-NEC trial (GETNE-T1913). Nat Commun. 2024;15(1):6753. 10.1038/s41467-024-50969-8 PMC11310219 39117670

[7] Borghesani M, Reni A, Lauricella E, Rossi A, Moscarda V, Trevisani E, et al. Efficacy and Toxicity Analysis of mFOLFIRINOX in High-Grade Gastroenteropancreatic Neuroendocrine Neoplasms. J Natl Compr Canc Netw. 2024;22(5):e247005 10.6004/jnccn.2024.7005 38744314

[8] Bodei L, Mueller-Brand J, Baum RP, Pavel ME, Hörsch D, O’Dorisio MS, et al. The joint IAEA, EANM, and SNMMI practical guidance on peptide receptor radionuclide therapy (PRRNT) in neuroendocrine tumours. Eur J Nucl Med Mol Imaging. 2013;40(5):800–16. 10.1007/s00259-012-2330-6 PMC3622744 23389427

[9] Hope TA, Abbott A, Colucci K, Bushnell DL, Gardner L, Graham WS, et al. NANETS/SNMMI Procedure Standard for Somatostatin Receptor-Based Peptide Receptor Radionuclide Therapy with (177)Lu-DOTATATE. J Nucl Med. 2019;60(7):937–43. 10.2967/jnumed.118.230607 PMC12079155 31263080

[10] Institute NC. Common Terminology Criteria for Adverse Events (CTCAE) Version 6.0. U.S. Department of Health and Human Services; 2025.

[11] Khoury JD, Solary E, Abla O, Akkari Y, Alaggio R, Apperley JF, et al. The 5th edition of the World Health Organization Classification of Haematolymphoid Tumours: Myeloid and Histiocytic/Dendritic Neoplasms. Leukemia. 2022;36(7):1703–19. 10.1038/s41375-022-01613-1 PMC9252913 35732831

[12] Arber DA, Orazi A, Hasserjian RP, Borowitz MJ, Calvo KR, Kvasnicka HM, et al. International Consensus Classification of Myeloid Neoplasms and Acute Leukemias: integrating morphologic, clinical, and genomic data. Blood. 2022;140(11):1200–28. 10.1182/blood.2022015850 PMC9479031 35767897

[13] Sabet A, Ezziddin K, Pape UF, Ahmadzadehfar H, Mayer K, Pöppel T, et al. Long-term hematotoxicity after peptide receptor radionuclide therapy with 177Lu-octreotate. J Nucl Med. 2013;54(11):1857–61. 10.2967/jnumed.112.119347 24009272

[14] Kwekkeboom DJ, de Herder WW, Kam BL, van Eijck CH, van Essen M, Kooij PP, et al. Treatment with the radiolabeled somatostatin analog [177 Lu-DOTA 0,Tyr3]octreotate: toxicity, efficacy, and survival. J Clin Oncol. 2008;26(13):2124–30. 10.1200/JCO.2007.15.2553 18445841

[15] Bergsma H, Konijnenberg MW, Kam BL, Teunissen JJ, Kooij PP, de Herder WW, et al. Subacute haematotoxicity after PRRT with (177)Lu-DOTA-octreotate: prognostic factors, incidence and course. Eur J Nucl Med Mol Imaging. 2016;43(3):453–63. 10.1007/s00259-015-3193-4 PMC4731438 26419852

[16] Svensson J, Berg G, Wängberg B, Larsson M, Forssell-Aronsson E, Bernhardt P. Renal function affects absorbed dose to the kidneys and haematological toxicity during ¹⁷⁷Lu-DOTATATE treatment. Eur J Nucl Med Mol Imaging. 2015;42(6):947–55. 10.1007/s00259-015-3001-1 PMC4382534 25655484

[17] Voter AF, Gafita A, Werner RA, De Jesus-Acosta A, Rowe SP, Solnes LB. Elevated Baseline Mean Corpuscular Volume Predicts the Development of Severe Hematologic Toxicity After (177)Lu-DOTATATE Therapy. J Nucl Med. 2024;65(9):1423–6. 10.2967/jnumed.124.267462 38991754

[18] Persson M, Hindorf C, Ardenfors O, Larsson M, Nilsson JN. Risk of treatment-altering haematological toxicity and its dependence on bone marrow doses in peptide receptor radionuclide therapy. EJNMMI Res. 2024;14(1):13. 10.1186/s13550-024-01077-7 PMC10847080 38319478

[19] Denoyer D, Lobachevsky P, Jackson P, Thompson M, Martin OA, Hicks RJ. Analysis of 177Lu-DOTA-octreotate therapy-induced DNA damage in peripheral blood lymphocytes of patients with neuroendocrine tumors. J Nucl Med. 2015;56(4):505–11. 10.2967/jnumed.114.145581 25722453

[20] Bodei L, Kidd M, Paganelli G, Grana CM, Drozdov I, Cremonesi M, et al. Long-term tolerability of PRRT in 807 patients with neuroendocrine tumours: the value and limitations of clinical factors. Eur J Nucl Med Mol Imaging. 2015;42(1):5–19. 10.1007/s00259-014-2893-5 25273832

[21] Kwekkeboom DJ, Krenning EP. Peptide Receptor Radionuclide Therapy in the Treatment of Neuroendocrine Tumors. Hematol Oncol Clin North Am. 2016;30(1):179–91. 10.1016/j.hoc.2015.09.009 26614376

[22] Desai P, Roboz GJ. Clonal Hematopoiesis and therapy related MDS/AML. Best Pract Res Clin Haematol. 2019;32(1):13–23. 10.1016/j.beha.2019.02.006 30927970

[23] Kusne Y, Patnaik MM, Halfdanarson TR, Sonbol MB. Therapy-related myeloid neoplasms in 177Lu-DOTATATE treated neuroendocrine tumor patients: how great is the risk? Endocr Relat Cancer. 2025;32(6):e250025 10.1530/ERC-25-0025 40446156

